# Supplementary material for: Amorphous Sb2S3 Nanospheres In-Situ Grown on Carbon Nanotubes: Anodes for NIBs and KIBs
Source: Nanomaterials (Basel). 2019 Sep 15;9(9):1323. doi: 10.3390/nano9091323 (PMC6781055; doi:10.3390/nano9091323)
Supplement: Supplementary file 1 [file nanomaterials-09-01323-s001.pdf]

nanomaterials

Supplementary

Amorphous Sb_2S_3 Nanospheres In-situ Grown on Carbon Nanotubes as Anodes for NIBs and KIBs

Meng Li [1], Fengbin Huang [1], Jin Pan [1], Luoyang Li [1], Yifan Zhang [1], Qingrong Yao [1], Huaiying Zhou [1], Jianqiu Deng [1,*]

[1] School of Materials Science and Engineering & Guangxi Key Laboratory of Information Materials, Guilin University of Electronic Technology, Guilin 541004, China; 185686394@qq.com (M.L.), 2664110462@qq.com (F.H.), 253309260@qq.com (J.P.), 1508321432@qq.com (L.L.), 479975825@qq.com (Y.Z.), qingry96@guet.edu.cn (Q.Y.), zhy@guet.edu.cn (H.Z.)

* Correspondence: jqdeng@guet.edu.cn (J. D.)

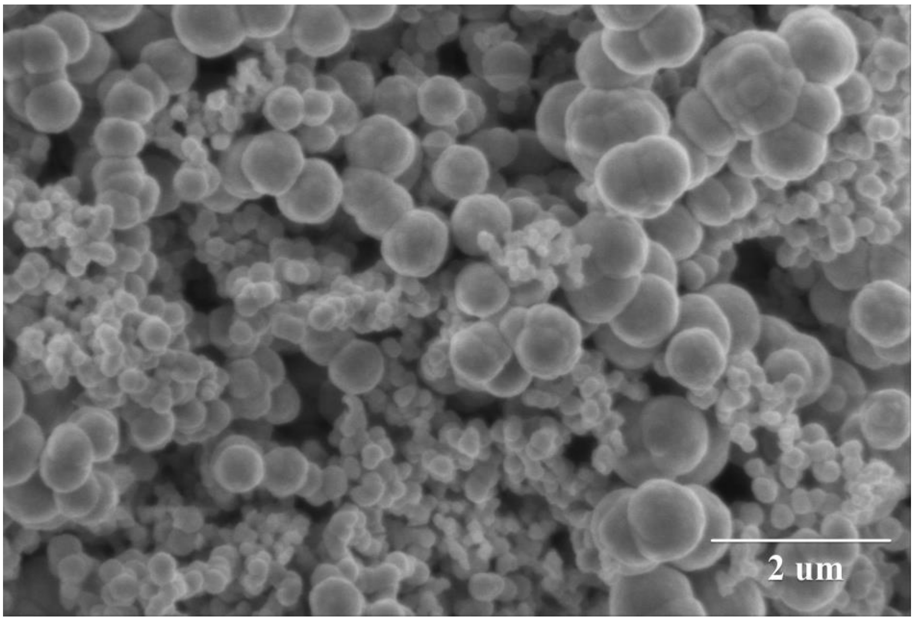

Figure S1. SEM image of pure Sb_2S_3.

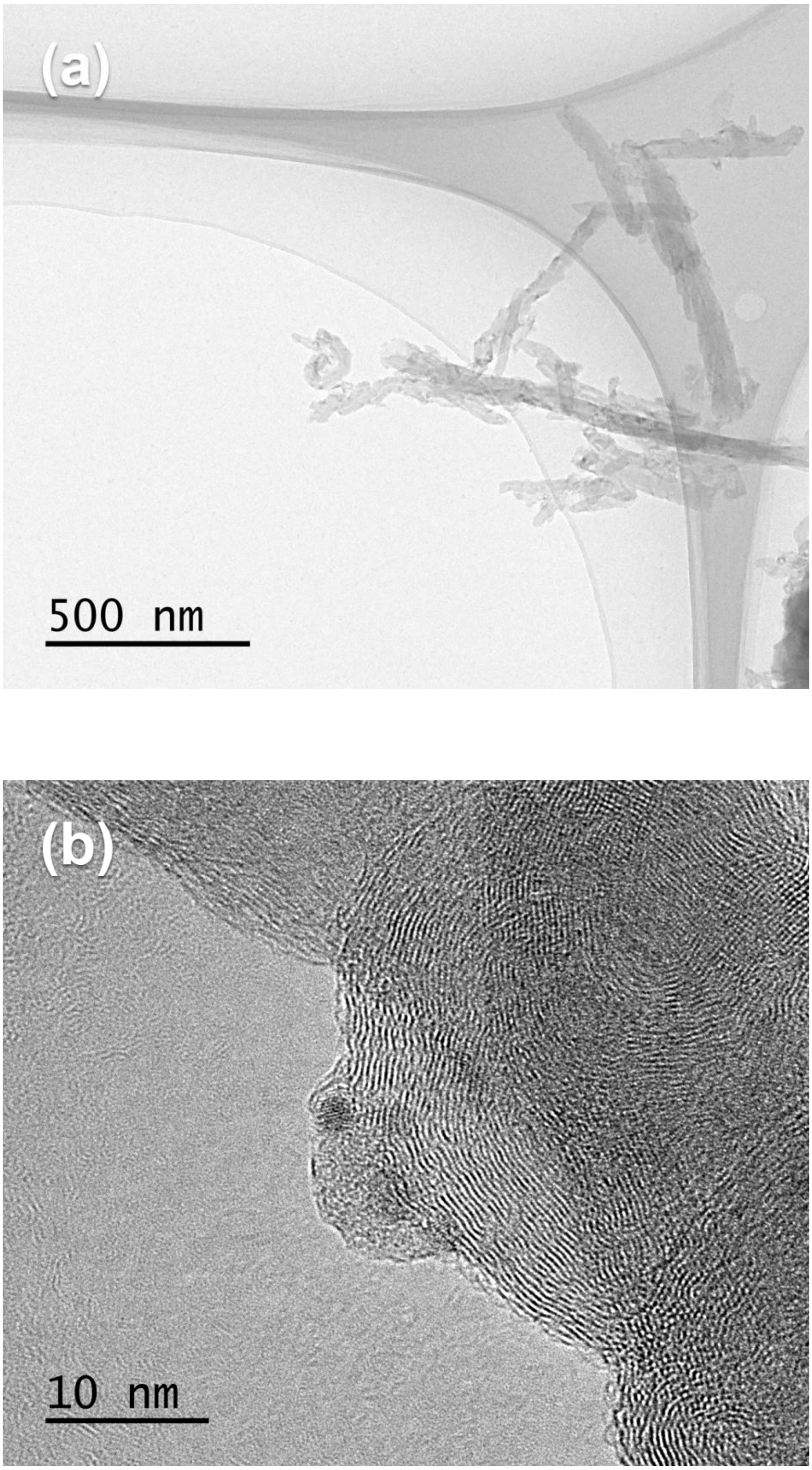

Figure S2. (a) TEM and (b) HRTEM images of the CNTs.

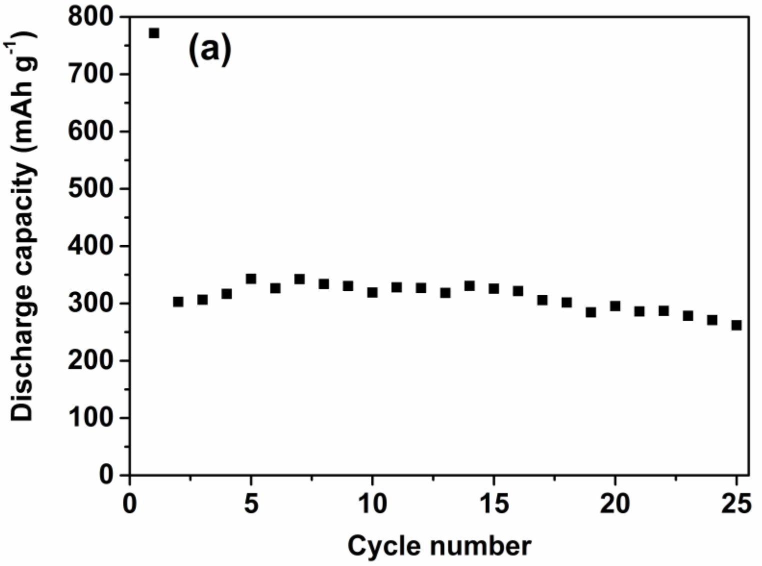

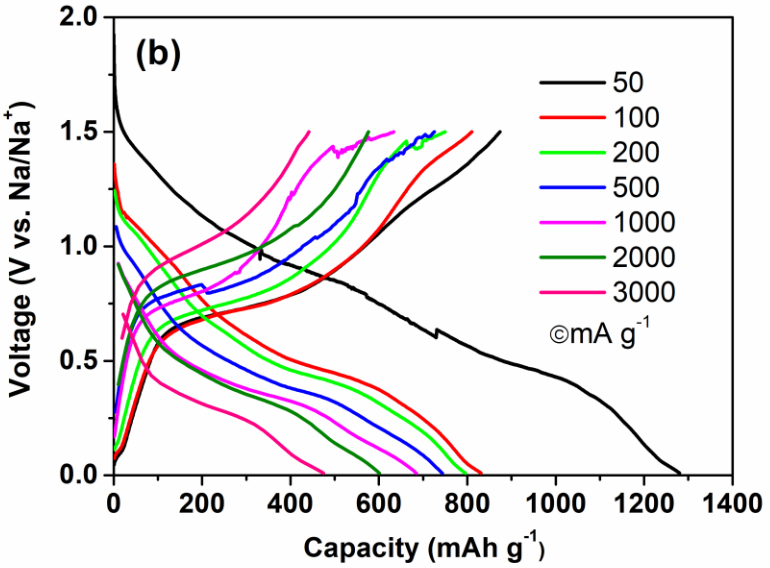

Figure S3. (a) Cycle performance of the Sb_2S_3 anode for NIBs at 100 mA g^{-1}. (b) Galvanostatic discharge–charge profiles of the Sb_2S_3/CNTs anode for NIBs measured under various current densities.

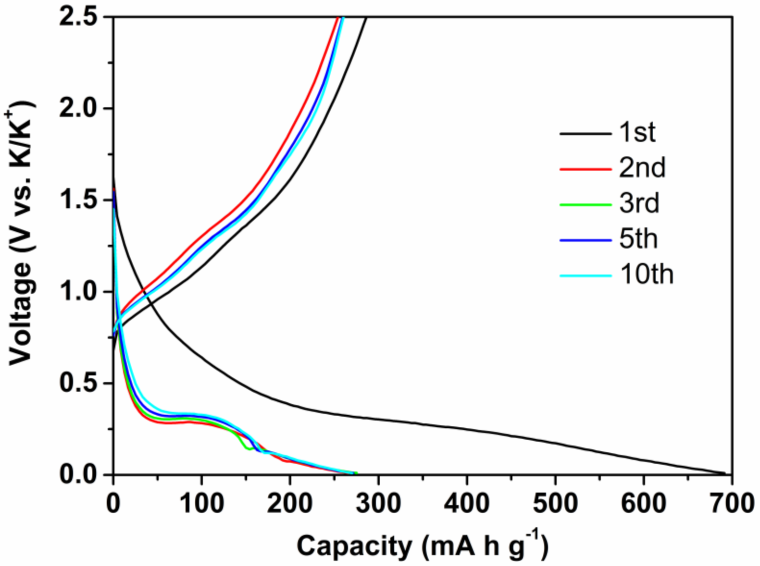

Figure S4. The galvanostatic discharge/charge curves of the Sb2S3/CNTs anode for KIBs at 500 mA g^{-1}.
